# Potentiating T cell tumor targeting using a combination of TCR with a Siglec-7 based CSR

**DOI:** 10.3389/fimmu.2025.1536868

**Published:** 2025-05-13

**Authors:** Shiran Didi-Zurinam, Erel Katzman, Cyrille J. Cohen

**Affiliations:** Laboratory of Tumor Immunology and Immunotherapy, The Goodman Faculty of Life Sciences, Bar-Ilan University, Ramat Gan, Israel

**Keywords:** T-cells, immunotherapy, co-stimulatory chimeric switch receptor (CSR), sialic acids, Siglec-7

## Abstract

**Introduction:**

Tumors may utilize different strategies to escape T cell immunosurveillance. Besides the overexpression of checkpoint ligands (such as PDL1) or the secretion of immunosuppressive agents, several studies have shown that cancer aberrant sialylation can, through interaction with selected receptors such as those from the Siglec family, neutralize NK and T cell function.

**Methods:**

Herein, we wanted to take advantage of the presence of inhibitory sialic acid ligands on the tumor cell surface to enhance T cell anti-tumor activity. To this end, we devised a novel chimeric receptor consisting of the extracellular portion of Siglec-7 and the intracellular portion of 41BB, which can convert inhibitory signals into stimulatory ones when expressed in human T-cells.

**Results:**

This co-stimulatory chimeric switch receptor (CSR), when co-expressed with a tumor-specific TCR, facilitated higher cytokine secretion and activation profiles following co-culture with tumor cells. Additionally, T cells equipped with Siglec-7 CSR demonstrated improved anti-tumor function *in vivo*.

**Discussion:**

Given the broad expression pattern of Siglec-7 ligands on tumor cells, our data suggest this CSR may act as a general adjuvant to boost TCR T cell function. Overall, this work provides an approach to improve engineered T-cell-based cancer treatment.

## 1 Introduction

Sialic acids are a diverse family of nine-carbon sugar molecules that are often positioned on the end of glycans of cell surface glycoproteins and glycolipids that play crucial roles in cellular processes, particularly in the modulation of immune responses and cell-cell interactions (1–3). Sialic acid residues can be bound to more than one terminal sugar, for example, via an α2,3- or α2,6-linked bond (4). It was demonstrated that these chemical compounds can often contribute to the regulation and dampening of the immune response. As such, sialic acids can be upregulated on the surface of tumor cells, through a process referred to as “hypersialylation” which facilitates their evasion of immune detection and promoting tumor progression (5, 6).

Tumor associated sialic acids negatively influence immune cell function by interacting with the sialic acid binding immunoglobulin-like lectin (Siglec) family (7). This family includes 14 Siglecs identified as functional in humans and 9 Siglecs in mice (8). Siglecs can be divided into two sub-groups, CD33-related Siglecs, and conserved Siglecs, based on sequence similarity and evolutionary conservation. The CD33-related Siglecs differ in composition between species, share high sequence similarity in their extracellular regions, and frequently contain conserved tyrosine-based signaling motifs in their intracellular domains (9). Depending on their intracellular signaling domains, Siglec receptors can also be classified into inhibitory, activating, and non-signaling Siglec receptors (10).

Siglec-7 is a natural killer (NK) cell-inhibitory receptor bearing ITIM motifs and is mainly expressed on NK cells, monocytes, macrophages, mast cells, neutrophils, dendritic cells, and a minor subset of CD8^+^ T cells (11–13). This receptor preferentially binds to α2,3- and α2,6-linked sialic acids and plays a role in downregulating cell activation signaling pathways, thereby modulating immune responses and contributing to immune evasion in cancer (14, 15). Siglec-7 is primarily involved in the negative regulation of the immune response, particularly in natural killer (NK) cells and T cells (11), where it inhibits their cytotoxic functions. This inhibition is mediated by the recruitment of SHP1/2 following the activation of ITIM motifs within Siglec-7 (16, 17). Previous studies have demonstrated that Siglec-7 ligands are broadly expressed across multiple solid tumors, including melanoma, glioblastoma, breast, and pancreatic cancers (18–21). Thus, Siglec-7 represents an attractive target for immunotherapeutic intervention.

Over the last decade, significant advancements in cancer therapy have been achieved through immunotherapy, including checkpoint inhibitors, tailored cancer vaccines, and adoptive cell transfer (ACT) with tumor-specific lymphocytes. Genetic modification of T cells to display new specificities can be achieved by introducing a T cell receptor (TCR) or a chimeric antigen receptor (CAR) specific for a defined antigen (22). One key difference between native T cell receptors (TCRs) and chimeric antigen receptors (CARs) is that CARs include co-stimulatory domains. To add co-stimulation to TCR T cells, one can co-express co-stimulatory molecules such as CD28 or 4-1BB (23, 24), provided their corresponding ligands are present on the target cells. Alternatively, we and others also showed that one may co-express chimeric co-stimulatory switch receptors (CSRs); these chimeric molecules combine the extracellular domain of an inhibitory receptors (for example, PD1, TIGIT) linked to the intracellular domain of costimulatory ones (25–27). CSRs were shown to increase T cell anti-tumor function and recently, their benefit was investigated in clinical trials (28, 29).

As sialic acids are widely expressed by tumor cells, we aimed to take advantage of these inhibitory ligands to enhance T cell anti-tumor activity. To this end, we sought to develop and characterize a Siglec-7-based CSR as a chimeric receptor composed of Siglec-7 and 41BB. We successfully achieved high expression levels of this chimeric receptor and demonstrated its enhancing capabilities by means of cytokine secretion and upregulation of activation markers. Finally, we showed in a xenograft mouse model of human tumors that S7-41BB can mediate tumor growth delay and enhanced survival.

## 2 Materials and methods

### 2.1 Patient PBMCs and cell lines

Peripheral blood mononuclear cells (PBMCs) were obtained from healthy donors from the Israeli Blood Bank (Tel-Hashomer, Israel). Melanoma cell lines 624.38 (HLA-A2^+^/MART-1^+^) and 888 (HLA-A2^−^/MART-1^+^) were generated at the Surgery Branch (National Cancer Institute, National Institutes of Health, Bethesda, MD). 888/A2 is an HLA-A2^+^ transduced line derived from 888. A375 (CVCL_0132) is a melanoma cell line which is HLA-A2^+^/MART-1^-^ used as negative control. The viral packaging line 293GP (CVCL_E072) stably expresses GAG and POL proteins. Adherent cells were cultured in DMEM (Sartorius, Germany), supplemented with 10% heat-inactivated FBS, 1% L-Glutamine, 1% Pen-Strep solution, and 0.01M HEPES. Human lymphocytes were cultured in a 1:2 mix of RPMI 1640 and TexMACS™ Medium (Miltenyi Biotec, USA), supplemented with 10% heat-inactivated FBS, 1% L-Glutamine, 1% Pen-Strep solution, 0.01M HEPES, and 300 IU/ml IL-2 (Peprotech, Israel). All cells were maintained at 37°C and 5% CO_2_.

### 2.2 TCR and Siglec chimera retroviral constructs

The retroviral vector backbone used in this study, pMSGV1, is a derivative of the MSCV-based splice-gag vector, which uses a murine stem cell virus (MSCV) long terminal repeat and was previously described (30). The α and β chains from the previously characterized TCR specific for MART-1 termed F4 (or DMF4) and the different Siglec-7 chimeras and full-length constructs were subcloned into the pMSGV1 vector as described previously (31). The Siglec-7-based chimeric receptor was created by overlapping PCR.

### 2.3 Antibodies and flow cytometry

Fluorophore-labeled antibodies against human Siglec-7, CD8, CD4, CD137, LAG3, CD69, CD25, CCR7, and CD45RO were purchased from BioLegend (San Diego, CA, USA). Cells were stained in a FACS buffer made of PBS, 0.5% BSA, and 0.02% sodium azide for 30 min at 4°C in the dark. Anti-Vβ12 antibody (Beckman Coulter Cat# IM2291, RRID: AB_131198, Marseille, France) is specific for F4 TCR. Staining of α2,6-linked or α2,3-linked sialic acids was done using FITC conjugated Sambucus Nigra Agglutinin (SNA) or Maackia amurensis agglutinin (MAL) respectively (Vector Laboratories, Burlingame, CA, USA). Siglec-7-Fc was purchased from R&D Systems (Minneapolis, MN). Cells were analyzed by flow cytometry, gated on the live population as described using a Cytoflex 6-colors apparatus (Beckman, Indianapolis, IN).

### 2.4 Cytokine release and cytotoxicity assays

The cytokine release measurements were preformed using commercially available human ELISA kits for IL-2, IFNγ, and TNFα (R&D Systems, Minneapolis, MN, USA). For these assays, 1x10^5^ T cells and 1x10^5^ tumor cells were incubated for 24 hours in 200 μL of culture media in individual wells of 96-well plates. For the cytotoxicity assay, 1x10^4^ mCherry expressing target cells were seeded on a flat bottom 96 plate well and co-cultured with T cells, at different Effector: Target (E:T) ratio for 48h in the IncuCyte^®^ Live-Cell Analysis System (Sartorius, Germany) and analyzed for the average orange integrated intensity of 3 replicates wells.

### 2.5 *In vivo* assay

NSG mice were inoculated with 1.5X10^6^ 888/A2 tumor cells in 100ul HBSS and 100µl Cultrex matrix (Trevigen), using an insulin syringe with a 27-gauge needle, in the dorsal flank of 6-12-week-old NSG mice. Upon tumor establishment, mice were randomized and injected into the tail vein with two injections of 5x10^6^ transduced lymphocytes on days 7 and 13 after tumor inoculation. There were no outliers. Tumor growth was measured every 2–3 days in a blinded fashion using a caliper and calculated using the formula: (Dxd2) x π/6, where D is the largest tumor diameter and d is its perpendicular diameter. All the procedures were approved by the university committee for animal welfare.

### 2.6 Statistical analysis

A paired *Student’s* t-test was used to determine statistical significance. Data are reported as mean ± SEM. Statistical values, including the number of replicates (n), can be found in the figure legends. ∗p < 0.05, ∗∗p < 0.01, ∗∗∗p < 0.001. Survival curves were compared using a LogRank analysis. The statistical test used for each figure is described in the corresponding legend.

## 3 Results

### 3.1 Design and expression of Siglec-7-based chimeric switch receptor

**Figure 1:**
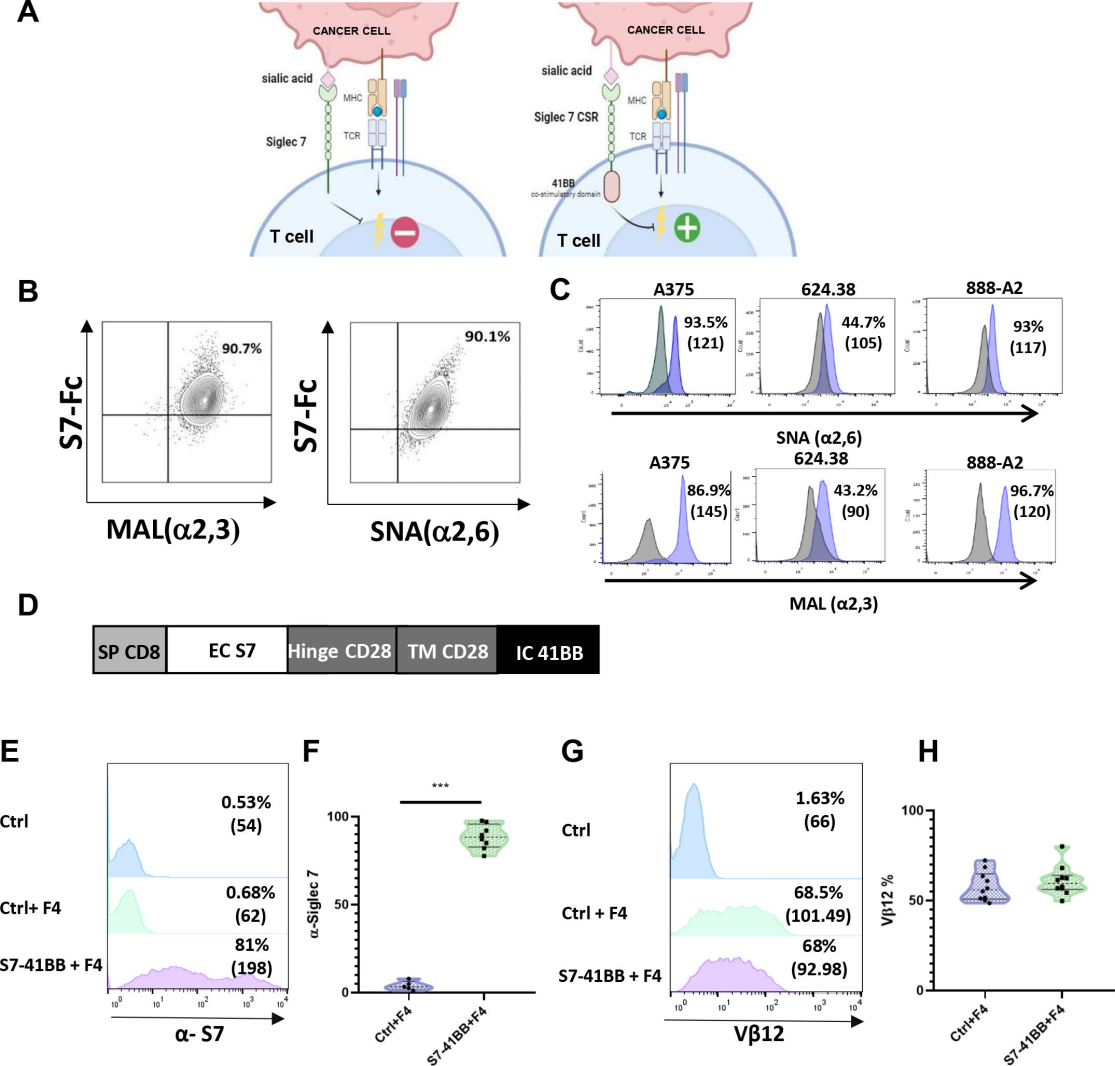
Design and Expression of Siglec-7-Based Chimeric Switch Receptor (CSR). **(A)** Schematic representation of Siglec-7 CSR function. Unlike endogenous Siglec-7, which transmits a co-inhibitory signal, the S7-41BB receptor in which the native intracellular domain was replaced by a signaling moiety derived from 41BB, conveys co-stimulation following the binding to sialic acid (designed by BioRender). **(B)** Siglec-7 binds to α2,3 and α2,6-linked sialic acid. We co-stained K562 cells with PE-labeled soluble Siglec7-Fc (S7-Fc) protein and either APC-labeled MAL or FITC-labeled SNA, which preferentially bind to sialic acid via α2,3 and α2,6 linkages, respectively, for 30 minutes on ice. The cells were then washed and analyzed by flow cytometry. **(C)** Tumor cell lines were stained with FITC-conjugated SNA to determine α2,6-sialic acid surface expression and APC-conjugated MAL to determine α2,3-sialic acid surface expression using flow cytometry. The grey histogram shows the unstained negative control, and the MAL or SNA-stained positive population is indicated in purple. The percentage of positive cells is indicated. **(D)** Structure of the Siglec-7 CSR: S7-41BB contains a CD8 SP domain, a native Siglec-7 extracellular domain, followed by CD28 hinge and transmembrane domains, and a 4-1BB intracellular moiety. **(E, F)** Human peripheral blood lymphocytes (PBLs) were transduced with a retroviral vector encoding S7-41BB or mock-transduced with an empty vector (Ctrl). 72 hours after transduction, transgene expression was measured by flow cytometry using an anti-Siglec-7 antibody. The left panel **(E)** shows a representative result, and the right panel **(F)** shows the mean ± SEM (***p<0.001; n=6 independent experiments, performed with at least 4 different donors). **(G, H)** In parallel, these cells were also transduced with the MART-1–specific F4 TCR. Representative flow cytometry histograms of F4 TCR expression were assessed by staining the cells with anti-vβ12 mAb. The left panel **(G)** shows a representative result, and the right panel **(H)** shows the mean ± SEM (n=6 independent experiments, with at least 4 different donors). The difference between the groups was not statistically significant (p=0.4; calculated using a *Student’s* paired t-test).

In the present study, we focused on CSRs based on the Siglec-7 receptor as a targeting moiety and the intracellular domain of the co-stimulatory molecule 4-1BB (**Figure 1A**). To detect the presence of Siglec-7 ligands on target cells, we utilized MAL and SNA lectins, which recognize sialic acid in α2,3 and α2,6 linkages (32). We confirmed their ability to recognize Siglec-7 ligands by co-staining K562 tumor cells with SNA+Siglec-7-Fc or MAL+Siglec-7-Fc, as shown in (**Figure 1B**). We further determined the extent of Siglec-7 ligand expression on several tumor cell lines, namely A375, 888/A2 and 624.38, and observed high levels of sialic acid domain (**Figure 1C**). Given the widespread presence of Siglec-7 ligands on tumor (18, 21, 33)and stromal cells (34), we hypothesized that designing an effective Siglec-7-based CSR should be relevant to the enhancement of engineered T cell anti-tumor response. We designed such a receptor, termed S7-41BB, by fusing Siglec-7 extracellular domain to the hinge and transmembrane region and a 41BB signaling domain (**Figure 1D**). Primary human T cells were transduced with this CSR and, in parallel, with MART-1 specific TCR F4 to generate tumor specificity. Flow cytometry analysis confirmed a high level of Siglec-7-based CSR surface expression, with 81% and an MFI of 198 positive cells compared to 0.68% and an MFI of 62 in the mock transduced control T cells (**Figures 1E, F**). Additionally, we confirmed similar TCR expression levels in both the control and the CSR (68-68.5%; **Figures 1G, H**), to negate any possible bias observed in T cell functionality due to inequivalent F4 expression.

### 3.2 Siglec-7-based CSR enhances T cell cytokine secretion and activation marker upregulation

**Figure 2:**
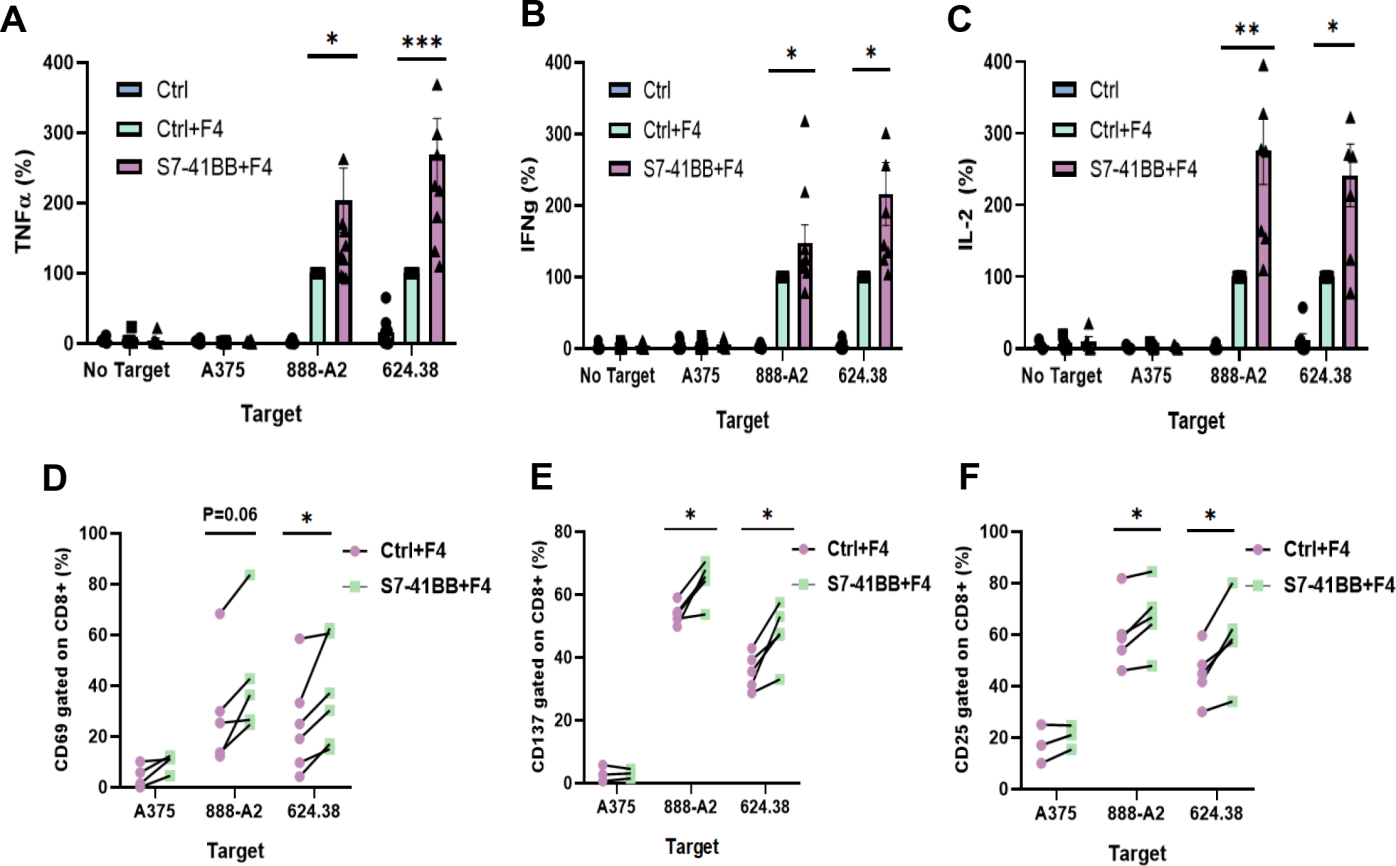
Siglec-7–based CSR can enhance TCR-engineered T cell function. **(A–C)** Primary human T cells were transduced with S7-41BB+F4 or with F4 TCR only (ctrl+F4). These cells were co-cultured overnight with melanoma tumor lines or without (“no target”), as indicated. TNFα **(A)**, IFNγ **(B)** and IL-2 **(C)** concentration secreted in the co-culture supernatant was measured by ELISA. These results are presented as mean + SEM (n = 6, with 3 different donors; normalized to the activity of positive control Ctrl+F4 against 888/A2 or 624.38). **(D–F)** Additionally, transduced T cells (either S7-41BB+F4 or Ctrl+F4) were co-cultured with different tumor lines as indicated for 4hr (for CD69) or overnight (for CD25 and CD137). After the co-culture, these cells were analyzed by flow cytometry for CD69 **(D)**, CD137 **(E)**, or CD25 **(F)** expression respectively, gated on the CD8+ population. The percentage of positive cells is shown (n=4–6 independent experiments performed with at least 3 different donors). The increase in activation marker expression was found to be statistically significant (*: p <0.05, **: p<0.01; ***: p<0.001, calculated using a *Student’s* paired t-test).

To assess the impact of Siglec-7-based CSR on T cell function, we first co-cultured engineered T cells with various human melanoma cell lines and measured TNFα, IFNγ and IL-2 secretion (**Figures 2A–C**). We observed a 1.5 to 2.8-fold increase in cytokine secretion by S7-41BB transduced T cell compared to TCR-only control, in co-cultures with 888/A2. Similarly, we observed an increase of 168% in TNFα, 116% in IFNγ and 142% in IL2 in co-cultures with the 624.38 cell line. No significant cytokine secretion was detected in co-cultures with MART1-negative control A375 or in the absence of targets. Overall, Siglec-7-based CSR enforced expression in T cells mediated an enhanced anti-tumor cytokine secretion capability.

We further assessed the upregulation of early (CD69) as well as late (4-1BB and CD25) markers of T cell activation. 4-1BB (CD137) facilitates T cells long-term survival and memory formation, CD25 is the α chain of IL-2 receptor, and CD69 is an early activation marker linked to T cell proliferation. Following co-culture with different tumor targets, we noted that Siglec-7-based CSR could trigger an upregulation of these markers compared to TCR-only control; for example, S7-41BB leads to 43% more expression CD69, 20% more 4-1BB, and 12% more CD25 expression in cocultures with 888/A2 (**Figures 2D–F**).

### 3.3 Phenotypic characterization of S7-41BB expressing T-cells

**Figure 3:**
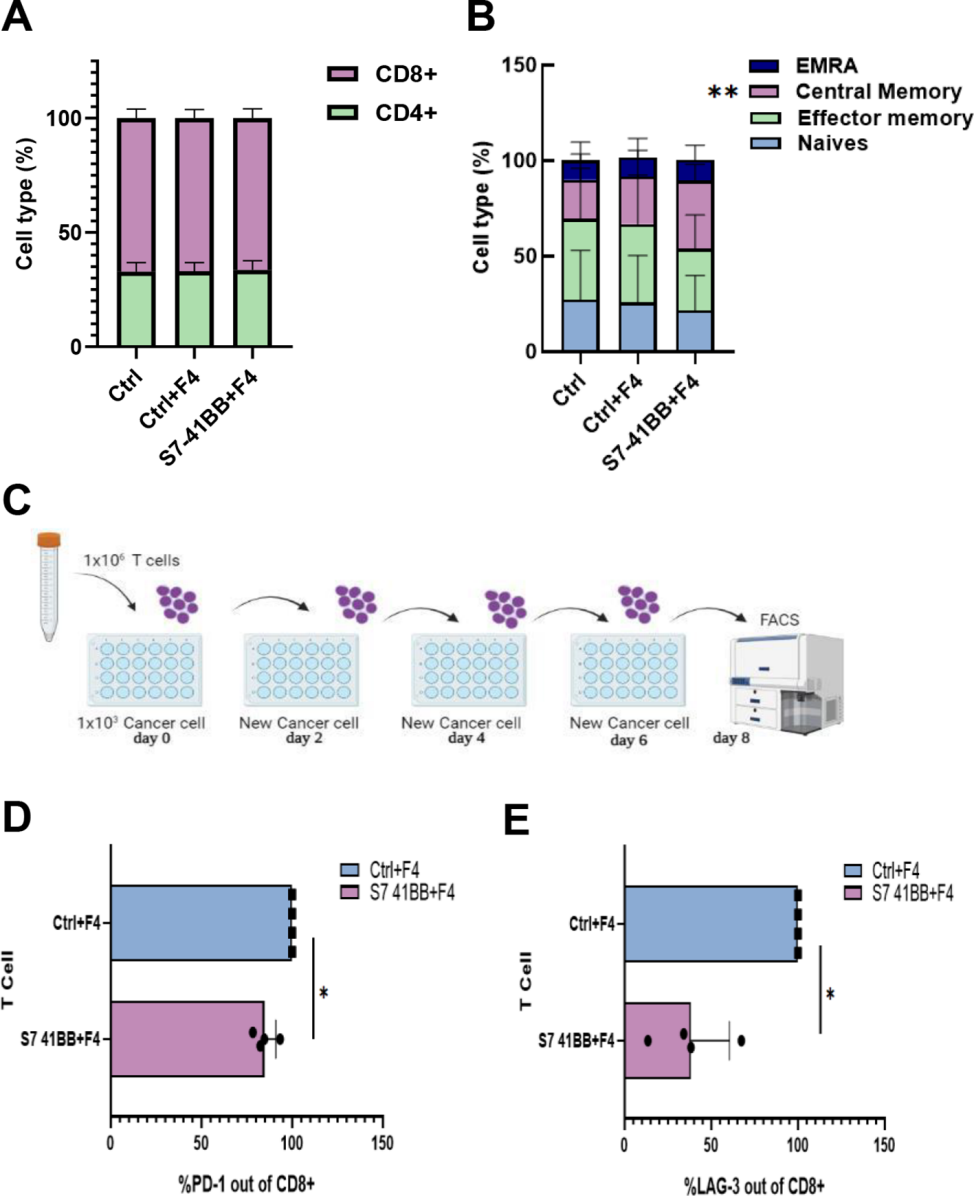
Siglec-7 CSR-based T cells show decreased expression of exhaustion markers. **(A)** The CD4/CD8 ratio of transduced cells was determined by flow cytometry. These results are representative of n=4 independent experiments with 4 different donors. No significant difference was observed between the Ctrl groups and S7- 41BB group. **(B)** The effector/memory phenotype of transduced cells was determined by flow cytometry based on CD45RO and CCR7 expression. EM—effector memory (CD45RO+/CCR7−), CM—central memory (CD45RO+/CCR7+), EMRA—terminally differentiated effector memory cells re-expressing CD45RA (CD45RO−/CCR7−) or naïve cell population (CD45RO−/CCR7+). These results are presented as the mean+SEM of n=5 independent experiments with 3 different donors. We found that only the percentage of central memory cells was statistically significant between Ctrl+F4 (control group) and S7- 41BB. (**p=0.01, using a *Student’s* paired t-test). **(C)** Schematic representation of the assay we developed to test T cell function after antigen re-exposure (designed by BioRender). **(D, E)** Transduced T cells with S7-41BB+F4 or Ctrl groups cells were co-cultured with 888/A2 melanoma tumor lines as indicated. After 3 or 8 days, these cells were analyzed by flow cytometry for expression of PD-1 or LAG-3 (respectively), gated on the CD8+ population. PD-1 **(D)** and LAG 3 **(E)** expressions are displayed. These results are representative of 3 independent experiments with 3 different donors and were found to be statistically significant (*: p< 0.05, calculated using a *Student’s* paired t-test).

Following the transduction of T cells with S7-41BB, we also measured the distribution of CD4^+^/CD8^+^ cells several days of culture. We did not observe a statistically significant difference in the CD4/CD8 ratio between the S7-41BB and control populations, with an approximate of ratio 30%/70% (**Figure 3A**). Similarly, we assessed the memory phenotype of these different populations by staining them for CD45RO and CCR7 expression and dividing them into effector memory, central memory, EMRA (terminally differentiated effector memory cell re-expressing CD45RA) or naïve cell population. A significant increase in the percentage of central memory T cells was observed in S7-41BB expressing cells compared to controls - 35.7% vs. 20.24% respectively (*p=0.01; **Figure 3B**).

In addition, we assessed the expression of PD-1 and LAG-3 exhaustion markers in a hypofunction induction assay by repetitively exposing T cells to tumor cells (**Figure 3C**). Indeed, PD-1 and LAG-3 are receptors that can, upon ligation to their ligands, downregulate T cell activity and proliferation (35–37). As seen in **Figures 3D, E**, Siglec-7-based CSR could trigger a downregulation of these markers; for example, S7-41BB leads to 15% less PD-1 expression, and 60% less LAG-3 expression (**Figures 3D, E**). Overall, Siglec-7-based CSR can mediate an increase in the central memory compartment and diminish the expression of immune checkpoint receptors.

### 3.4 4-1BB intracellular domain is essential to Siglec-7 CSR function in T cells

**Figure 4:**
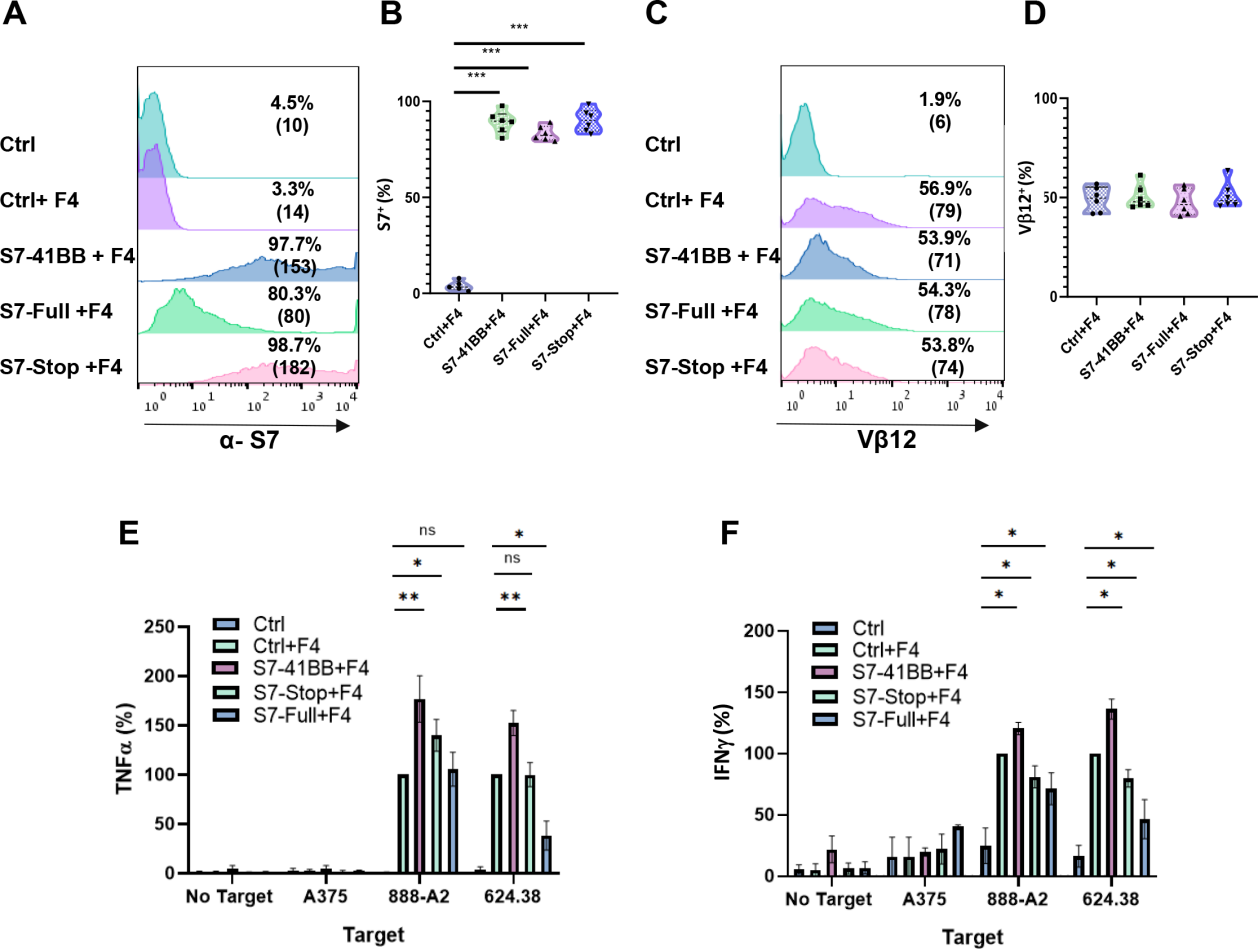
Expression and impact of S7-STOP on Cytokine Secretion in T Cells. **(A, B)** Human PBLs were transduced with a retroviral vector encoding Ctrl, S7-41BB, S7-Full, S7-Stop. 72h after transduction, transgene expression was measured by flow cytometry using antibodies specific for Siglec-7. The left panel **(A)** is a representative result, and the right **(B)** panel shows the mean+SEM of n=6 independent experiment performed with at least 4 different donors. The difference between Ctrl+F4 and each of the transduced cell population with a different Siglec-7 construct was found significant (***p<0.001; using a *Student’s* paired t-test). **(C, D)** These cells were transduced also with the MART-1–specific F4 TCR. Representative flow cytometry histograms of F4 TCR expression were assessed by staining the cells with an anti-vβ12 mAb. The left panel **(C)** is a representative result, and the right panel **(D)** shows the mean+SEM of n=6 independent experiment performed with at least 4 different donors. The difference between the groups population was not found statistically significant (calculated using a *Student’s* paired t-test). **(E, F)** Transduced T cells were co-cultured with melanoma tumor lines or without (“no target”), as indicated. After 24 hours, the supernatants were analyzed by ELISA for secretion of TNFα **(E)** and IFNγ **(F)**. Cytokine secretions were normalized to that from the TCR-only group (Ctrl + F4) against each target cell line and are represented as the mean+SEM (*n* > 4; * *P* < 0.05, ** *P* < 0.01 calculated using a *Student’s* paired t-test). ns, not significant.

We sought to demonstrate the importance of the 4-1BB co-stimulatory intracellular domain of the CSR. Thus, we generated two additional constructs: Siglec-7-Stop, a truncated receptor which lacks the native intracellular domain and Siglec-7-Full, the native Siglec-7 molecule. We transduced T cells with both F4 TCR and these constructs or S7-41BB (**Figures 4A–D**) and co-cultured them with melanoma targets. As seen in **Figure 4E**, in co-cultures with 888/A2, S7-41BB mediated an improvement of 76% in TNFα secretion, in comparison to S7-Stop (which failed to meaningfully improve cytokine secretion), excluding the possibility that S7-41BB CSR acts as a decoy receptor. Interestingly, we noted that T cells overexpressing the full-length Siglec-7 receptor demonstrated a 30-50% reduction in IFNγ secretion in co-cultures with melanoma cell lines (*p<0.05; 624.38 and 888/A2; **Figure 4F**), suggesting that Siglec-7 may fulfill an inhibitory function in primary human T-cells.

### 3.5 Siglec-7-based CSR improves T cell anti-tumor function *in vitro* and *in vivo*

**Figure 5:**
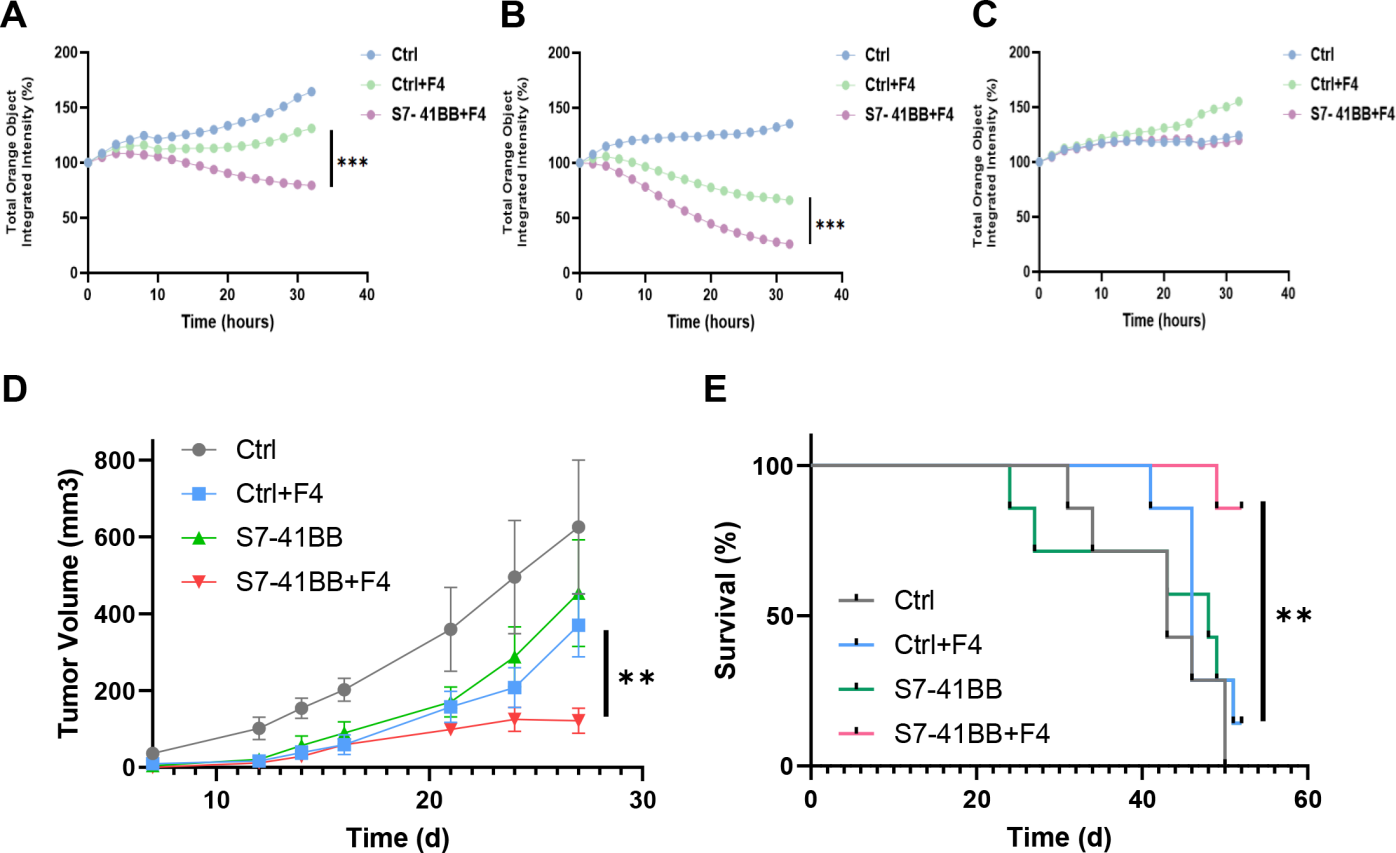
Siglec-7–based CSR mediates significant cytotoxic activity. Siglec-7–based CSR demonstrates an antitumor response *in vivo*. **(A–C)** S7-41BB+F4 or Ctrl+F4-transduced T cells were co-cultured with the indicated target cell lines for 32 hours at an effector: target of ratio of 1:1 and 2:1. The total integrated intensity of mCherry fluorescence was measured to monitor the number of live cells and was normalized to *t* =0. These results are presented as the mean+ SEM of at least 3 independent experiments with 3 different donors. (A: A375 negative control line (1:1), B: 888/A2 mCherry (1:1) C:888/A2 mCherry (2:1)). **(D, E)** NSG mice were inoculated with 1.5x10^6^ tumor cells. Then, mice were injected with Ctrl, Ctrl+F4, S7-41BB S7-41BB+F4 transduced T cells. Two injections were performed on day d7 and d13 after tumor inoculation, with 5x10^6^ T cells. **(D)** Tumor volume was measured in a blinded fashion using a caliper and calculated using the following formula: (Dxd2) xπ/6, in which D is the largest tumor diameter and d is its perpendicular one. The average tumor volume of each treatment group (n=7) was measured over time and the difference was found statistically significant (**p= 0.008 using a *Student’s* t-test). **(E)** The survival percentage per treated group was determined and plotted. The difference between the S7-41BB+F4 or Ctrl+F4 groups was found to be statistically significant (**p=0.0063 using a Log Rank test). "***" = p < 0.001, as defined in Section 2.6.

To further examine the function of Siglec-7-based CSR on T cells, we conducted a cell-mediated cytotoxicity assay, evaluating live melanoma target cells following a 32-hour co-culture with T cells at various Effector: Target (E:T) cell ratios (**Figures 5A–C**). Enhanced cytotoxicity was observed for CSR-transduced cells at 1:1 and 2:1 ratio. In **Figure 5A**, a decrease in the number of viable 888/A2 cells was observed after 32 hours at a 1:1 ratio, with only 79% viability of the target tumor cells in the S7-41BB+F4 group compared to 131% in the Ctrl+F4 group. Similar results were obtained at E:T ratio of 2:1, with a significant reduction of live target tumors (from 66% to 26%; ***p=0.001) between the control and the S7-41BB+F4 group respectively (**Figure 5B**). No significant cytotoxicity activity was observed against the A375 cell line (**Figure 5C**).

Finally, we assessed the *in vivo* anti-tumor function of Siglec-7-based CSR T cells and their ability to influence tumor growth in a human tumor xenograft mouse model. For this purpose, 1.5x10^6^ tumor cells (888/A2) were injected into the flank of NSG mice. 5x10^6^ T cells (Ctrl, Ctrl+F4, S7-41BB or S7-41BB+F4) were injected through the tail vein, one and two weeks after tumor cell injection. We monitored tumor growth and observed that S7-41BB+F4 T cells delayed tumor growth compared to the control group treated with Ctrl+F4-transduced T cells (**Figure 5D**; n=7, p=0.008). Furthermore, at the experiment endpoint, 85% of the mice treated with Siglec-7-based CSR survived compared to 14% in the control group (**Figure 5E**; **p=0.0063). In conclusion, Siglec-7-based CSR T cells could delay tumor growth and significantly prolong the survival of tumor-bearing mice.

## 4 Discussion

Adoptive T cell transfer-based immunotherapies for cancer have demonstrated remarkable advancements with the implementation of engineered T cell treatments. Still, efficacy remains limited, especially when targeting solid tumors (22, 38). In that regard, we and others have shown that chimeric switch receptors (CSRs) significantly enhance the anti-tumor activity of T cells. Some of the previous CSR designs relied on checkpoint ligands, such as PD-L1 or CD155, which are not always consistently expressed in tumor cells (25, 26, 39–41). Siglec ligands can be broadly expressed through “hypersialylation” on the surface of approximately 50% of tumor cells (including lung, breast, ovarian, pancreatic, and prostate cancers) (5, 6, 35). Thus, we aim to develop CSRs targeting Siglec-7 as an effective strategy to enhance cellular immunotherapy.

Siglec-7 is considered an inhibitory receptor in immune cells such as lymphocytes or myeloid cells (42–44). Consistently, we observed that overexpressing full-length Siglec-7 in T cells reduced cytokine secretion (**Figure 3**), reinforcing its putative role as an inhibitory checkpoint (11). Alternatively, we show that following the replacement of the intracellular inhibitory domain with a costimulatory signaling moiety (4-1BB), we were able to significantly improve anti-tumor function. Indeed, S7-41BB-expressing T cells demonstrated enhanced cytokine production and upregulation of activation markers when co-cultured with melanoma cells, indicating a more robust anti-tumor response. Phenotypic characterization revealed a relative increase in central memory T cells and decrease in exhaustion markers, suggesting the possibility to achieve improved persistence and long-term anti-tumor activity while potentially counteracting T cell exhaustion. Moreover, *in vivo* xenograft studies presented herein provide evidence for the therapeutic potential of this approach.

As there are several molecules able to convey co-stimulatory signals in immune cells, one may envisage assessing the function of Siglec-7 CSRs with additional co-stimulatory moieties CD28, OX40, TLR domains (45–47), or even designing 2^nd^ generation CSR that may encompass several co-stimulatory domains in tandem. Nonetheless, recent findings suggest that 4-1BB-based CSRs exhibit superior activity compared to CD28-based designs (21). Still, we plan to evaluate Siglec-7 CSRs incorporating CD28 and OX40, with the aim of further optimizing this approach for distinct tumor microenvironments in future studies. We have shown that CSR function is dependent on specificity receptors activating T cells (known as “signal 1”) (48, 49). This is evidenced by the fact that antigen negative targets cells (A375) did not stimulate cytokine secretion (**Figure 2**), even in the presence of a high level of sialic acid ligands (**Figure 1C**). Thus, this design limits off-tumor effects by ensuring that the Siglec-7 CSR requires concurrent TCR activation even if sialic acids are widely expressed on normal tissues. Future studies could evaluate whether Siglec-7 CSRs exhibit any unintended interactions with healthy cells expressing high levels of sialylation, particularly in non-tumor immune compartments. Strategies such as fine-tuning receptor affinity or incorporating safety switches may help mitigate potential bystander effects while maintaining anti-tumor efficacy. Although, in this study, signal 1 was induced using a melanoma specific TCR, we suggest that Siglec-7 CSR may be assessed in conjunction with TCRs targeting other antigens and/or CARs, enabling the combination of different costimulatory signaling domains or a “if-better” signal (50). Additionally, CSRs may be utilized to increase avidity, as has been recently demonstrated (51) and increase the sensitivity to the antigen.

Further optimization of Siglec-7 chimeras could focus on the targeting moiety. Indeed, it has been shown that Siglec-7 comprises three Ig-like domains, with domains 1 and 3 being essential for its function (33). This suggests that a more compact and optimized CSR might be developed using only these critical domains. Moreover, while this study primarily focused on Siglec-7 as a targeting moiety, other Siglec molecules, such as Siglec-9, Siglec-10 or Siglec-15, could also be explored as potential targeting moieties. These receptors exhibit differential binding preferences for tumor-associated sialylation patterns and may provide additional avenues to optimize glyco-immune checkpoint targeting. Future studies could investigate the relationship between the effectiveness of Siglec-7-based CSR T cells and the degree of tumor sialylation, with the goal of identifying predictive markers to select suitable patients. Since Siglec-7 ligands are present on both glycoproteins and glycolipids, it would be valuable to determine whether CSRs behave differently depending on the type of residue they bind to, or whether the nature of the sialic acid linkage (α2,3, α2,6, or α2,8) may affect the CSR function.

The potential applications of Siglec-7-based CSRs may reach beyond cancer therapy (52, 53). Given that Siglec receptors can detect sialoglycan ligands on cells infected by viruses like HIV, HBV, and SARS-COV2 (54–56), there is a possibility that Siglec-7-based CSRs could enhance the performance of T cells engineered with virus-specific TCRs. This suggests another potential avenue for expanding the use of this technology to combat persistent viral infections.

Nonetheless, several limitations and questions remain to be addressed. While CSRs cannot function without an additional activation signal provided for example by a TCR, further studies will be needed to assess the long-term safety and efficacy of this approach, including potential off-tumor effects given the presence of sialic acids on normal tissues (57). Additionally, combining this strategy with other immunotherapeutic approaches, such as checkpoint inhibitors or other engineered receptors, could potentially yield synergistic benefits and warrants investigation.

In conclusion, this study presents a novel strategy to enhance the anti-tumor function of engineered T cells by exploiting tumor-associated sialic acids. This Siglec-7-based CSR shows promise as a versatile tool to improve T cell-based immunotherapies, potentially addressing key challenges in the field such as T cell exhaustion and tumor immune evasion. Further research and clinical development of this approach could lead to more effective T cell-based treatments for a broad range of cancers.

## Data Availability

The raw data supporting the conclusions of this article will be made available by the authors, without undue reservation.
